# 卤化多肽真空紫外激光解离及蛋白质组学分析

**DOI:** 10.3724/SP.J.1123.2024.08009

**Published:** 2025-02-08

**Authors:** Pan LUO, Jieying XUE, Zheyi LIU, Fangjun WANG

**Affiliations:** 中国科学院大连化学物理研究所,中国科学院分离分析化学重点实验室,辽宁大连116023; CAS Key Laboratory of Separation Sciences for Analytical Chemistry, Dalian Institute of Chemical Physics, Chinese Academy of Sciences, Dalian 116023, China

**Keywords:** 蛋白质组学, 光化学卤化, 串联质谱, 193 nm紫外激光解离, 自由基解离, proteomics, photochemical halogenation, tandem mass spectrometry (MS/MS), 193 nm ultraviolet photodissociation (193 nm UVPD), radical dissociation

## Abstract

化学修饰在蛋白质组学定量和相互作用分析等研究领域应用广泛。本工作发展了一种针对复杂组织提取蛋白质酶解样品的多肽溴化、碘化光化学修饰新方法,实现了酪氨酸、组氨酸、色氨酸位点的高效溴化和酪氨酸、组氨酸位点的高效碘化修饰。进一步采用193 nm真空紫外激光解离(UVPD)串联质谱技术对光化学溴化和碘化标记后的鼠肝酶解肽样品进行了序列、修饰位点和光解离机制分析。由于Br和I原子对193 nm紫外光子的强吸收特性,193 nm UVPD可引起卤化位点的C-Br/C-I键断裂并产生多肽自由基离子,自由基迁移进一步引起多肽骨架碎裂。此外,193 nm UVPD与常规的高能碰撞诱导解离(HCD)模式结合可以提升蛋白质组学的卤化位点定位准确性。因此,整合光化学卤化修饰和193 nm UVPD可引发新型自由基解离途径,提升蛋白质组学的分析性能。

## Abstract (en)

Chemical modifications are widely used in research fields such as quantitative proteomics and interaction analyses. Chemical-modification targets can be roughly divided into four categories, including those that integrate isotope labels for quantification purposes, probe the structures of proteins through covalent labeling or cross-linking, incorporate labels to improve the ionization or dissociation of characteristic peptides in complex mixtures, and affinity-enrich various poorly abundant protein translational modifications (PTMs). A chemical modification reaction needs to be simple and efficient for use in proteomics analysis, and should be performed without any complicated process for preparing the labeling reagent. High reaction specificity, which reduces product complexity, and mild biocompatible reaction conditions are also favored. In addition, modification labels should be compatible with mass spectrometry to prevent interference from ionization and dissociation processes. Pulsed ultraviolet (UV) lasers can produce large amounts of active radical species within a few nanoseconds for use in rapid photochemical-modification processes. Usually, UV lasers with wavelengths greater than 240 nm are used in current in-situ photochemical-modification methods; consequently, special conjugated photoreaction probes need to be designed and oxidants and catalysts added, which reduce the biocompatibility of the reaction. The high single-photon energy of the 193 nm laser is capable of efficiently exciting conventional photo-inert substances in aqueous solution, leading to efficient photochemical peptide modifications. In this study, we developed a new method for photochemically brominating and iodinating enzymatic protein samples extracted from complex tissue with a 193 nm ArF nanosecond pulsed laser, which efficiently brominated tyrosine, histidine, and tryptophan, and iodinated tyrosine and histidine.

Tandem mass spectrometry (MS/MS) can generate fragmentation patterns of ions which can afford diagnostic molecular fingerprints to decipher sequences of biopolymers such as peptides. Peptide fragmentation is commonly implemented using collision-based, electron-based, or photodissociation-based methods. Compared with the most commonly used collision-based methods, ultraviolet photodissociation (UVPD) uses high-energy ultraviolet photons with wavelengths shorter than 200 nm to excite and dissociate ions. Single-pulse excitation can provide the energy required to promote ions into their excited electronic states, with excitation speeds of up to several nanoseconds. Since dissociation may occur directly from the excited states, UVPD spectra can show a wide variety of fragmentation pathways, thereby providing more sequence and structural information. The most commonly used wavelengths are 157, 193, and 266 nm. UVPD has been integrated into high-resolution orbitrap mass spectrometer by adding optical windows and other optics to direct the photons to the analyte ions, and by implementing a triggering method that synchronizes the photoirradiation process with ion-analysis events. The large photoabsorption cross sections of peptides at 193 nm and the resulting high internal energy deposition can generate abundant fragment ions and achieve high sequence coverage. The excellent fragmentation performance offered by 193 nm UVPD of peptides with its high sequence coverage and lack of charge-state dependence, has motivated its use in high-throughput proteomics. Photochemically brominated and iodinated mouse-liver tryptic peptides were further characterized by 193 nm UVPD tandem mass spectrometry with the aim of analyzing their sequences, modification sites, and photodissociation mechanisms. Br and I atoms strongly absorb 193 nm photons; consequently, UVPD can cleave C-Br/C-I bonds at halogenated sites to generate peptide radical ions, with further peptide-backbone fragmentation caused by radical migration. In addition, the combination of 193 nm UVPD with conventional high-energy collision-induced dissociation (HCD) mode improves the identification-reliability of halogenation sites in proteomics. Therefore, integrating photochemical halogenation and 193 nm UVPD can trigger novel radical-dissociation pathways, thereby improving analytical proteomics performance.

蛋白质组学是全面分析生物体内蛋白质组成、功能和相互作用的学科,高分辨质谱是蛋白质组学研究的核心技术。除了通用的蛋白质组学工作流程,利用化学修饰引入标签可以显著促进质谱法对蛋白质组的全面解析。化学修饰的目标可以大致分为4类:引入同位素标签进行定量;通过共价标记或交联来探测蛋白质的结构;引入标签以改善复杂混合物中特征肽段的离子化或解离特性;亲和富集各种低丰度的翻译后修饰(protein translational modifications, PTMs)^[1]^。在蛋白质组学分析中化学修饰方法应满足以下标准:反应简单高效、无需复杂的标记试剂制备过程;高反应特异性以降低样品的复杂性和假阳性;温和的反应条件以保留非共价相互作用和结构信息;标签与质谱分析兼容,防止干扰离子化和解离过程。常规>240 nm的光化学修饰方法通常需要设计光化学探针并添加各种催化剂、氧化剂,而193 nm脉冲光化学标记方法无需各种添加剂,可在纳秒尺度实现肽段的高效光化学溴化和碘化修饰。

基于质谱的蛋白质组学分析高度依赖蛋白质或肽段离子的高效解离,以获取丰富的序列和结构信息^[2]^。质谱解离模式可以分为碰撞激活、电子激活和光激活3种类型^[3]^。碰撞激活是目前最常用的二级质谱解离方法,主要包括碰撞诱导解离(collision-induced dissociation, CID)和高能碰撞诱导解离(high energy collision dissociation, HCD),被广泛应用于商用串联质谱仪中。反应机理是通过与非反应性气体原子之间的碰撞将部分动能转变为内能实现肽段离子的解离,主要断裂酰胺键产生b/y碎片离子,该方法的最大缺陷是易产生不稳定翻译后修饰(如磷酸化和糖基化)的中性丢失而影响肽段骨架的高效解离^[4]^。基于电子激活的质谱解离方法,例如电子捕获解离(electron capture dissociation, ECD)和电子转移解离(electron transfer dissociation, ETD),主要断裂酰胺键后的N-C键产生c/z碎片离子,避免翻译后修饰的中性丢失,但是对前体离子电荷态具有显著依赖性^[5]^。光解离通过气相离子吸收光子激发产生碎片离子,解离过程可根据激光参数的变化进行调节,目前已在多种波长下实现了气相离子的质谱解离,主要包括红外多光子解离(infrared multiphoton dissociation, IRMPD)和紫外激光解离(ultraviolet laser dissociation, UVPD)。UVPD通过吸收高能紫外光子选择性激发肽段骨架或特征标签基团,可在纳秒甚至更低的时间尺度激发和解离蛋白质离子,由于解离可能直接从激发态产生,可以观察到广泛多样的碎裂途径,从而获得高序列覆盖度和丰富的结构信息且有助于翻译后修饰的保留^[6]^。

在UVPD解离中最常使用的激光波长是157、193或266 nm。准分子激光器因产生的193 nm紫外激光可选择性激发肽段骨架,且在空气中传输损耗较小,已成功应用于蛋白质组学研究中,包括表征磷酸化^[7]^、硫酸化^[8]^和糖基化修饰位点^[9]^,也被成功应用于完整蛋白质和蛋白质复合物的结构分析^[10,11]^,可实现对蛋白质序列、翻译后修饰及结构的同时高精度解析。近年来,我们实验室致力于真空紫外激光解离质谱新仪器研制和相关应用研究,在蛋白质序列和结构分析中取得了系列进展^[12⇓⇓⇓-16]^。193 nm UVPD在蛋白质组学中的应用研究证明了其可实现蛋白质序列鉴定覆盖率和可靠性的同时显著提升^[17]^。通过化学修饰肽段引入光切割标签可实现氨基酸选择性的质谱解离。Julian等^[18]^结合4-碘苯甲酸修饰肽段和266 nm UVPD开发了一种自由基解离促进位点选择性键断裂的混合质谱策略,芳香族基团可吸收266 nm光子进而引发光不稳定性C-I键高效和高选择性均裂生成自由基物种,随后经碰撞活化进一步碎裂产生特殊诊断碎片离子。

本研究通过光化学标记方法实现了鼠肝酶解肽酪氨酸、组氨酸、色氨酸位点的高效溴化和酪氨酸、组氨酸位点的高效碘化修饰(图1a),并通过193 nm UVPD/HCD高分辨率质谱平台对光化学修饰后的鼠肝多肽样品进行了串联质谱分析。193 nm UVPD可引发卤化位点的C-Br键或C-I键断裂产生多肽自由基离子,并通过自由基迁移导致多肽骨架进一步碎裂产生Br、I丢失的碎片离子(图1b)。组学分析发现193 nm UVPD结合常规的HCD解离模式可以提高修饰位点的定位准确性。本研究发展了一种光化学溴化、碘化修饰和193 nm UVPD高效整合的新型蛋白质组学质谱分析策略,193 nm紫外激光在真空条件下引发了新型自由基解离模式,实现了卤化位点定位精度的同时提升。

**图 1:**
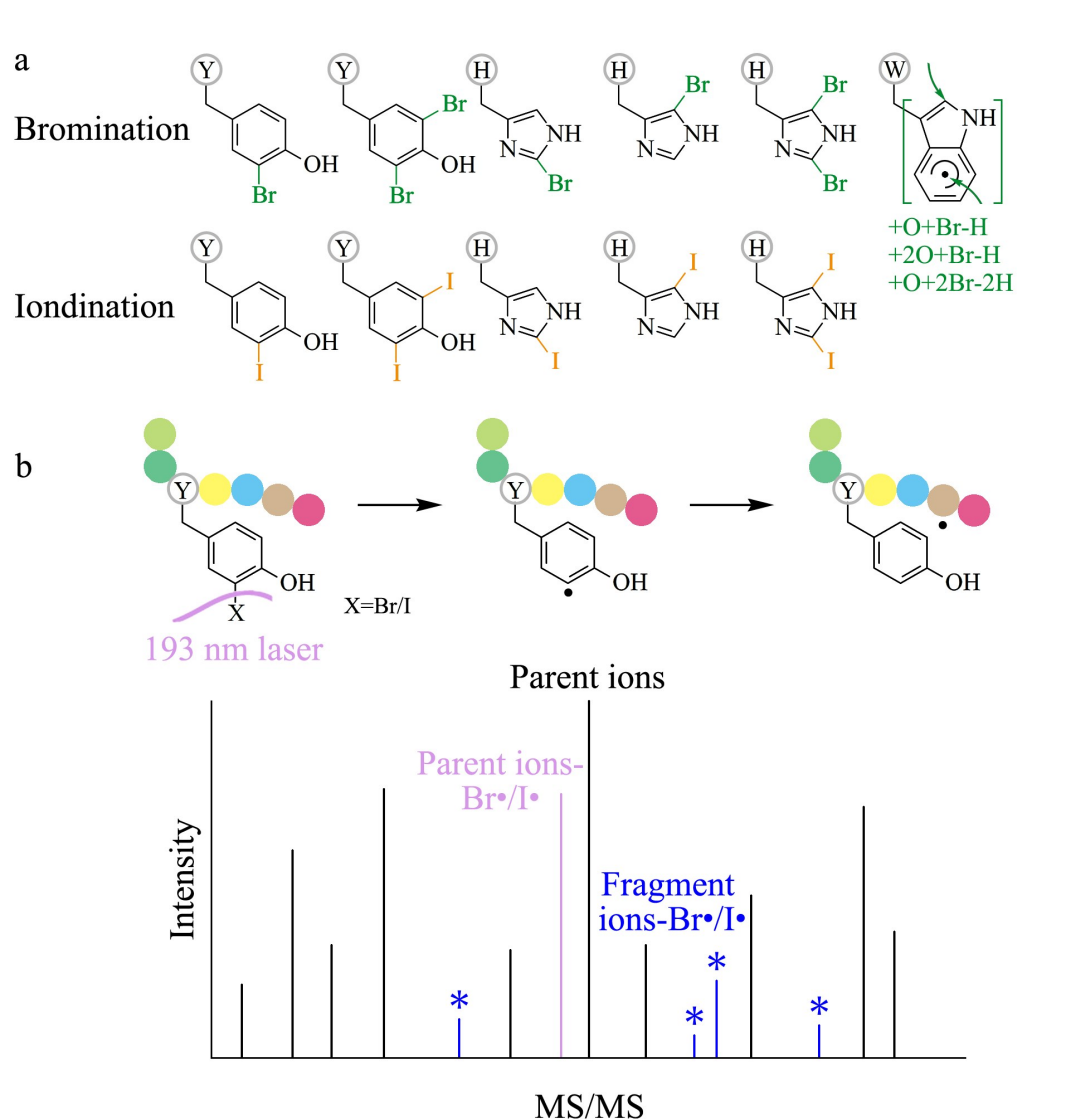
(a)酪氨酸、组氨酸和色氨酸的主要卤化产物以及(b)卤化肽段的193 nm UVPD解离示意图

## 1 实验部分

### 1.1 仪器、试剂与材料

Vanquish Flex超高效液相色谱系统和Orbitrap Fusion Lumos Tribrid三合一超高分辨质谱分析系统(Thermo Fisher,美国); 193 nm ArF准分子激光器(Coherent Laser Systems Gmbh & Co. KG,德国)。

甲醇(色谱纯)和乙腈(色谱纯)购自Merck公司(德国)。牛血清白蛋白(BSA)、胰蛋白酶(trypsin)、三(2-羧乙基)膦(TCEP)、碘乙酰胺(IAA)、BCA(二辛可宁酸)试剂盒、甲酸(FA)、三氟乙酸(TFA)、溴化钠(NaBr)、碘化钠(NaI)、磷酸一氢钠(Na_2_HPO_4_)、磷酸二氢钠(NaH_2_PO_4_)、尿素、三羟甲基氨基甲烷盐酸盐(Tris-HCl)、十二烷基硫酸钠(SDS)、蛋白酶抑制剂cocktail和其他未经标注的化学试剂均购自Sigma-Aldrich公司(美国)。熔融石英毛细管购自Polymicro Technologies公司(美国)。C18 AQ beads(3 μm, 12 nm)购自Sunchrom公司(德国)。实验中所用到的去离子水均由Milli-Q纯水系统(美国)制备。鼠肝组织严格按照欧盟动物管理规范(1986)的要求从大连医科大学获取。

### 1.2 鼠肝酶解肽和BSA酶解肽制备

将老鼠肝脏切成小块,用预冷的PBS缓冲液冲洗3次后悬浮于自制的裂解液(含8 mol/L尿素、50 mmol/L Tris-HCl、2% SDS和1% cocktail, pH 8.0)中混合均匀。在冰浴中进行超声破碎(200 W, 5 s×5 s×30次),并在4 ℃下超速离心(20000 g, 30 min)去除碎片。最后加入5倍体积的蛋白质沉淀液(丙酮-乙醇-乙酸,50∶50∶0.1, v/v/v)来提取蛋白质并在-20 ℃下保存过夜。在4 ℃下超速离心(20000 g, 30 min)收集蛋白质,并用预冷好的乙醇洗涤。鼠肝蛋白样品复溶于缓冲液中(含8 mol/L尿素和100 mmol/L NH_4_HCO_3_, pH 8.0)并通过BCA法测定蛋白质样品的浓度。10 mg蛋白样品分别用TCEP还原和IAA烷基化。然后,通过添加4倍体积的100 mmol/L NH_4_HCO_3_来稀释蛋白质溶液。再通过添加了质量比1∶50的酶-蛋白质的胰蛋白酶在37 ℃下过夜酶解。最后酶解肽利用C18 SPE柱除盐,冻干,存储于-80 ℃冰箱中待用。

### 1.3 紫外光化学溴化和碘化标记

鼠肝酶解肽样品以1 mg/mL的质量浓度溶于磷酸盐缓冲液(含10 mmol/L Na_2_HPO_4_、10 mmol/L NaH_2_PO_4_和150 mmol/L NaBr/NaI, pH=7.4)中并使用实验室之前搭建的单脉冲毛细管反应器进行光化学溴化和碘化标记^[19,20]^。标记完成后的鼠肝酶解肽样品通过C18 SPE柱除盐后冻干存储于-80 ℃冰箱中,等待进行后续的LC-MS/MS分析。

### 1.4 LC-MS/MS分析

从-80 ℃冰箱中取出鼠肝酶解肽样品,以0.1 μg/μL的质量浓度复溶于0.1% FA水溶液中。

将10 μL样品上样到自制的3 cm×150 μm i.d.的C18(3 μm, 12 nm)预柱上,再使用15 cm×75 μm i.d.的C18(3 μm, 12 nm)分析柱以200 nL/min的流速进行分离;流动相A相为0.1% FA水溶液,B相为0.1% ACN水溶液,分离梯度为B相在70 min内从5%升到35%。

在正离子模式下采集数据。一级质谱参数:电喷雾电压为2.3 kV,离子传输毛细管温度为275 ℃, Orbitrap分辨率为120000。二级质谱使用HCD和193 nm UVPD两种解离模式,具体参数:在HCD解离模式中,Orbitrap中以“top-speed”方式(3 s)采集,自动增益控制(AGC)为3×10^4^,最长离子注射时间为22 ms,分辨率为15000,解离的归一化碰撞能量(NCE)为28%;在UVPD解离模式中,193 nm激光能量1.5 mJ,脉冲为1 pulse,二级碎片离子同样在Orbitrap中以“top-speed”方式(3 s)采集,激发时间为1 ms, AGC为3×10^4^,最长离子注射时间为54 ms,分辨率为15000。

### 1.5 质谱数据分析

实验采集的raw文件数据在Proteome Discoverer 2.4软件中进行进一步数据库检索分析。检索参数设置如下:氨甲酰甲基化(carbamidomethylation, C)被设置为固定修饰;光化学溴化标记样品中Br1(YH)+77.9 Da、Br2(YH)+155.8 Da、Br1O1(W)+93.9 Da、Br2O1(W)+171.8 Da、Br1O2(W)+109.9 Da和Oxi(MW)+15.99 Da被设置为可变修饰;光化学碘化标记样品中I1(YH)+125.9 Da, I2(YH)+251.8 Da和Oxi(M)+15.99 Da被设置为可变修饰;a、b、c、x、y和z系列碎片离子权重值均被设置为1。结果与讨论部分的193 nm UVPD二级图谱均进行了y轴放大处理以更加清晰地展示产生的碎片离子峰。

## 2 结果与讨论

### 2.1 新型自由基解离模式

通过实验室之前开发的光化学溴化和碘化修饰方法可在温和的实验条件下对复杂鼠肝组织提取蛋白质酶解多肽的特定芳香和芳杂环氨基酸进行修饰从而引入独特的光切割标签,溴化标记的位点为酪氨酸、组氨酸、色氨酸,碘化标记的位点为酪氨酸、组氨酸^[20]^。266 nm光子已被证实在气相条件下可引发碘化酪氨酸和组氨酸的C-I键均裂产生自由基离子,随后进行碰撞活化以产生诊断片段离子^[18]^。文献报道Br、I原子在193 nm处具有强紫外吸收^[21,22]^,因此具有更高单光子能量的193 nm紫外光子在气相条件下有望引发溴化和碘化肽段修饰位点的C-Br/C-I键断裂产生肽段自由基。实验通过电喷雾离子化将光化学溴化和碘化标记后的鼠肝酶解肽转移到气相条件中,并基于同时配备193 nm UVPD和常规HCD两种解离模式的高分辨质谱平台对溴化和碘化标记后的鼠肝多肽样品进行蛋白质组学LC-MS/MS分析和数据库检索。

如图2所示,为了考察溴化修饰肽段的193 nm UVPD解离机制,实验选取了分别含有溴化酪氨酸、组氨酸、色氨酸位点的3条肽段VVEIAPATH(Br)LDPQLR、KPEEVDDEVFY(Br2)SPR、APM(O)FSW(BrO)PR进一步分析其碎片离子峰。对数据库中未匹配到的碎片离子通过单同位素质量手动匹配发现,母离子经193 nm UVPD解离后均产生了与Br丢失对应的质谱峰。将结果与同一峰的HCD解离图谱进行比较,证实了通过C-Br键的直接解离形成了Br丢失的肽段自由基。在HCD解离图谱中没有观察到母离子的Br丢失,可以证明C-Br键的裂解与266 nm下的C-I键的裂解相似,均通过区别于常规碰撞解离模式的高能解离途径进行^[18]^, C-Br键的光解离也必须从解离的激发电子态迅速发生。

**图 2:**
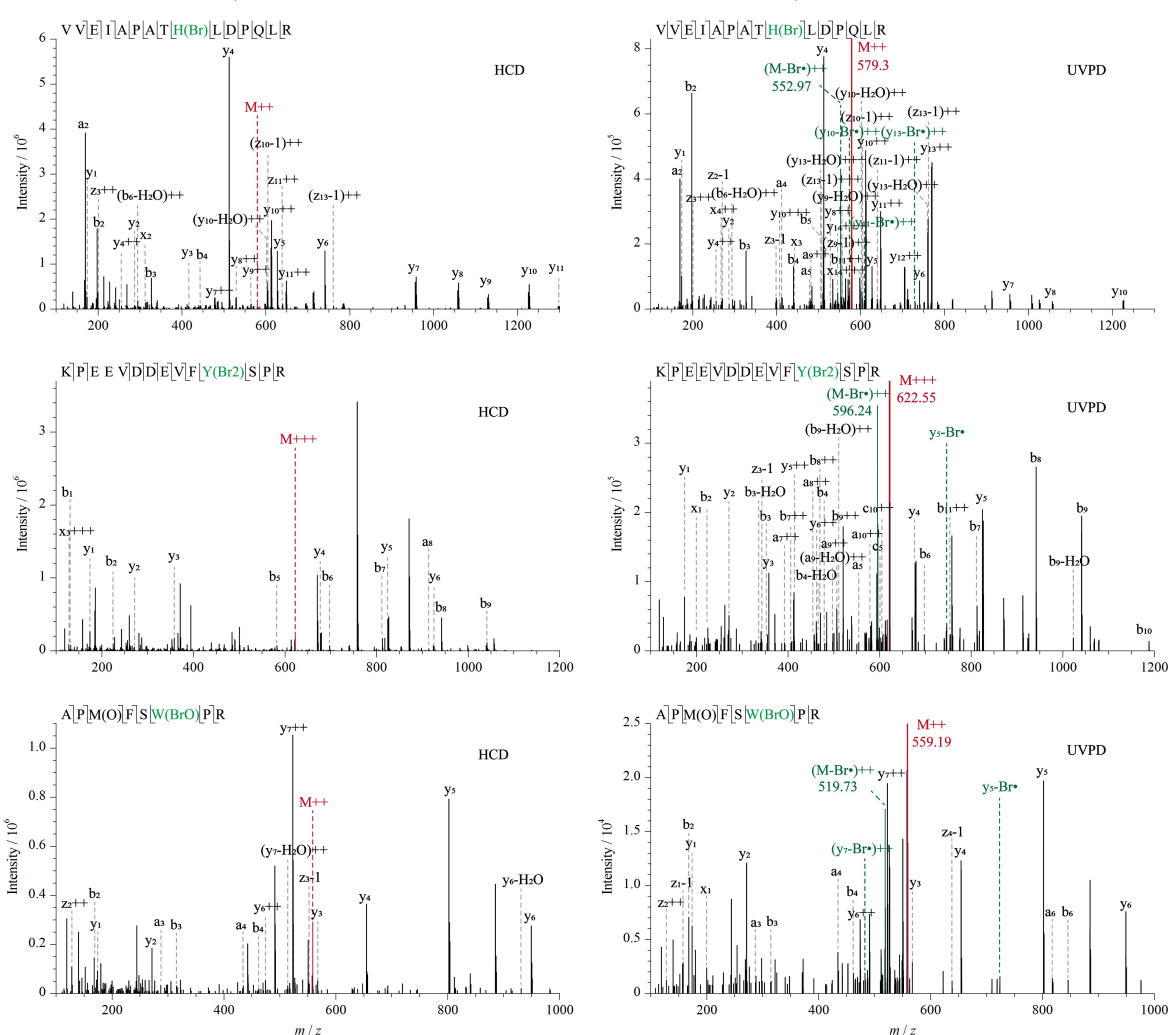
HCD和193 nm UVPD解离鉴定到的溴化肽段二级质谱图

如图3所示,进一步对含有碘化酪氨酸、组氨酸位点的两条肽段GVSEIVH(I)EGK、AADTIGY(I)PVMIR的二级图谱分析以考察碘化修饰肽段的193 nm UVPD解离机制。母离子经193 nm UVPD解离后均产生了与I丢失对应的质谱峰,且在同一峰的HCD解离图谱中没有观察到母离子的I丢失,证实了193 nm紫外光子引发了C-I键的直接解离形成了I丢失的肽段自由基。

**图 3:**
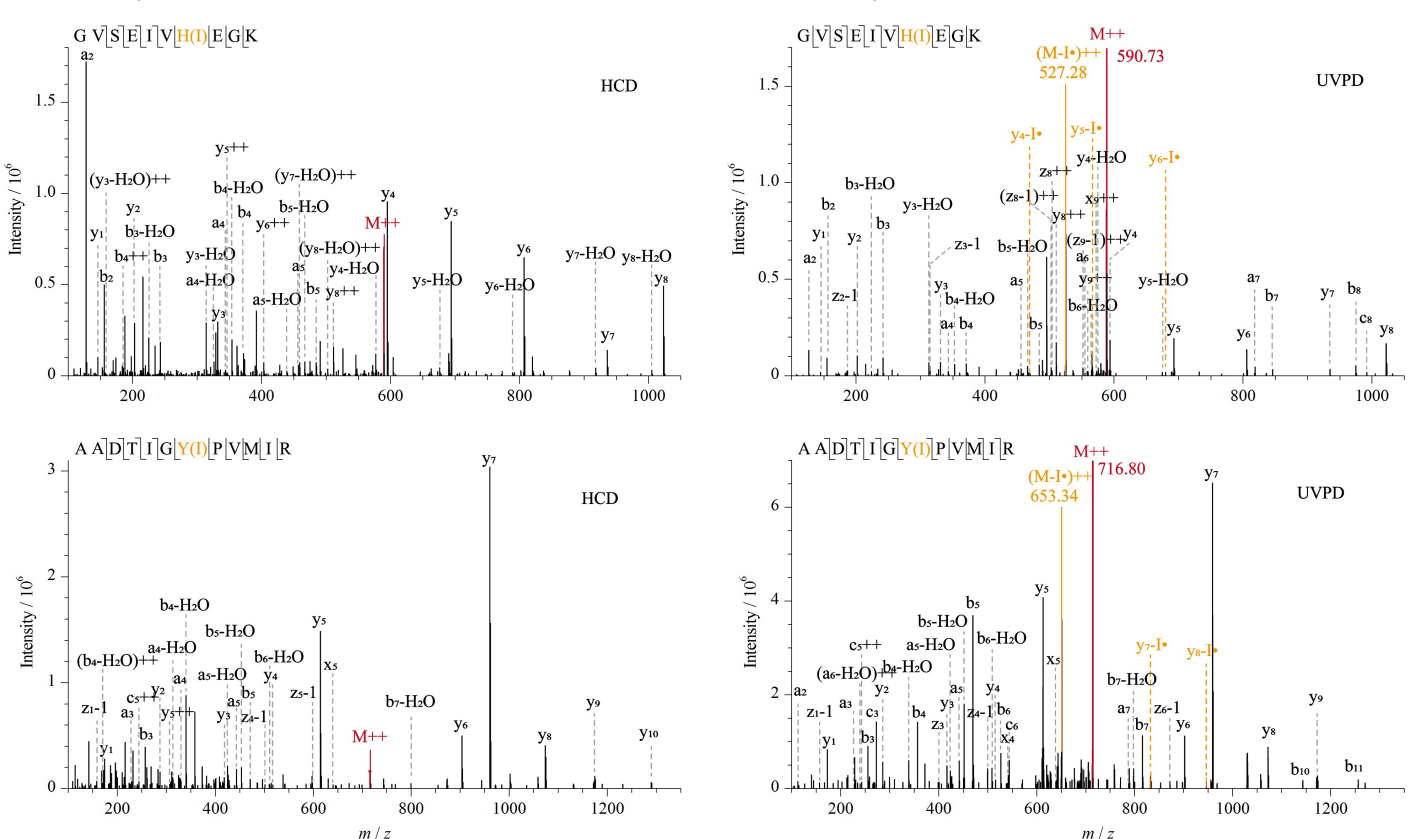
HCD和193 nm UVPD解离鉴定到的碘化肽段二级质谱图

此外,在肽段溴化和碘化修饰后的193 nm UVPD二级谱图发现了一些明显的Br、I丢失的y系列碎片离子峰:VVEIAPATH(Br)LDPQLR中的y_10_-Br·、y_11_-Br·、y_13_-Br·, KPEEVDDEVFY(Br2)SPR中的y_5_-Br·, APM(O)FSW(BrO)PR中的y_5_-Br·、y_7_-Br·, GVSEIVH(I)EGK中的y_4_-I·、 y_5_-I·、y_6_-I·, AADTIGY(I)PVMIR中的y_7_-I·、 y_8_-I·。芳香族C-Br键的解离能约为351 kJ/mol, C-I键的解离能约为280 kJ/mol, 193 nm紫外光子的能量相比266 nm光子更高,约为620 kJ/mol^[18]^。上述结果证明高能193 nm紫外光子激发卤化肽段到激发电子态,进而通过区别于常规碰撞解离模式的高能解离途径导致溴化酪氨酸、组氨酸、色氨酸的C-Br键断裂和碘化酪氨酸、组氨酸的C-I键断裂。这种光活化过程类似于266 nm光子引发的碘化酪氨酸和组氨酸的C-I键断裂,是一种选择性产生自由基位点的有效方法,然后肽段自由基的迁移引发了多肽骨架断裂产生了Br、I丢失的碎片离子。

### 2.2 卤化位点定位准确性提升

卤化修饰位点的精确定位需要依赖于修饰残基两侧骨架裂解所产生的N端和C端碎片离子的包围与标识。在对复杂组织样品酶解肽进行蛋白质组学分析时,常规的HCD解离模式可以实现大多数卤化肽段修饰位点的精确定位,但由于肽段数量众多,仍存在修饰位点定位不精确甚至于错误定位的情况。193 nm UVPD作为一种补充解离模式可一定程度提高卤化位点的定位准确性,这两种解离模式的结合可以提高蛋白质组学的分析性能。

以单碘化修饰肽段DIVEAHYR为例,肽段上有两个相邻的可修饰组氨酸和酪氨酸位点,要实现单碘化修饰的精确定位需要组氨酸和酪氨酸残基两侧骨架裂解的N端和C端碎片离子的包围与标识。

如图4所示,对比肽段的HCD和193 nm UVPD二级谱图发现,193 nm UVPD相比于HCD解离模式产生了更丰富的碎片离子数量并具有更高的打分(Xcorr)值。在HCD解离图谱中仅匹配到了y_1_和y_3_碎片离子峰,在数据库检索结果中自动将该肽段的碘化修饰位点定位到了组氨酸残基,导致了碘化位点的定位错误(图4a)。相比之下,在该肽段的193 nm UVPD解离图谱中额外鉴定到了y_2_碎片离子峰,y_1_的单同位素质量没有碘修饰的偏移,而y_2_相比未修饰的碎片离子峰偏移了一个碘的质量(图4b)。这表明DIVEAHYR肽段的单碘化修饰发生在酪氨酸而不是组氨酸残基,实现了碘化修饰位点的精确定位。此外,在193 nm UVPD图谱中也匹配到了I丢失的肽段自由基质谱峰和y系列碎片离子峰。

**图 4:**
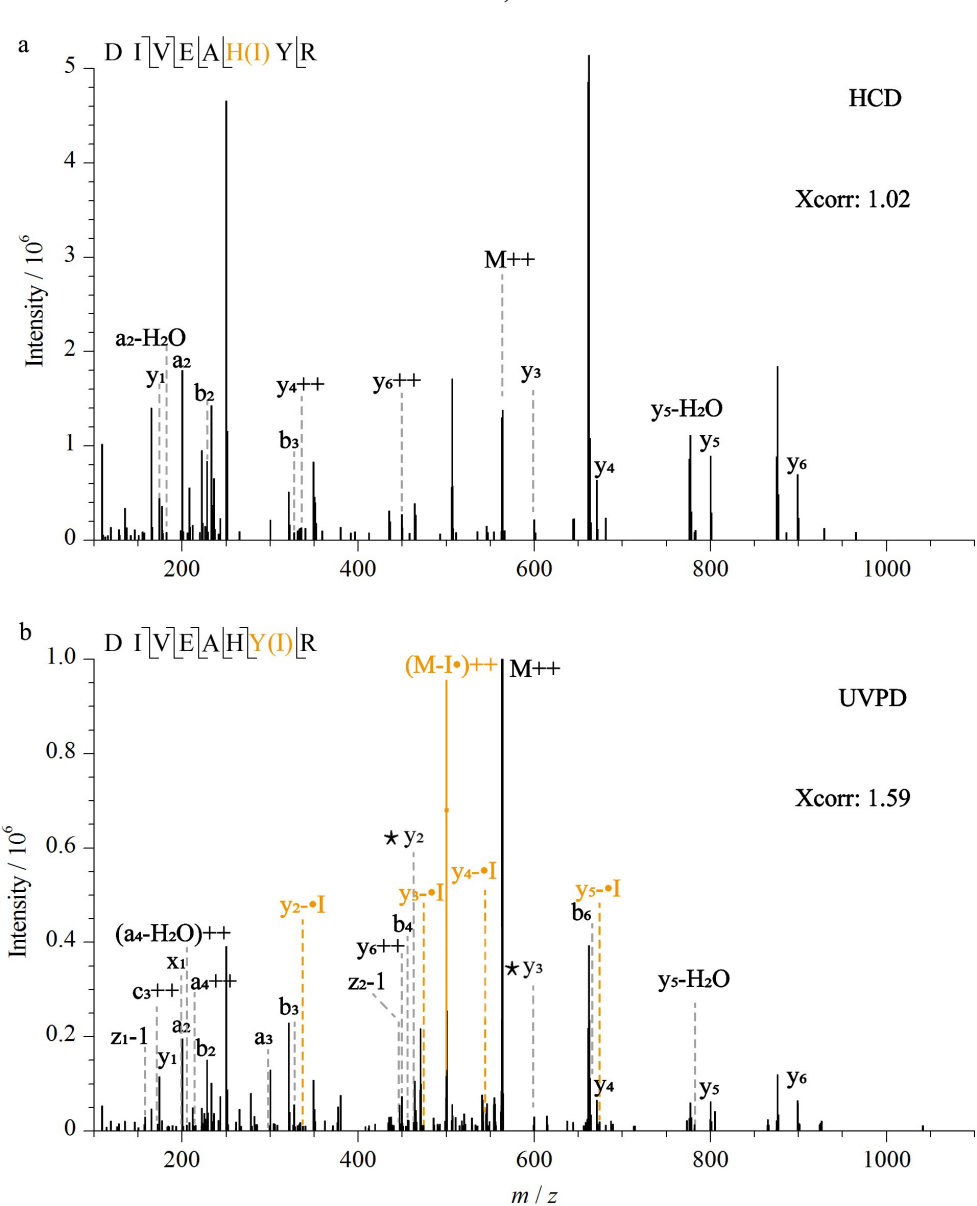
碘化DIVEAHYR肽段经(a)HCD和(b)193 nm UVPD 解离模式鉴定的二级质谱图

肽段SLGQWLQEEK根据一级质谱中母离子的单同位素质量匹配表明发生了溴氧化修饰,对于这种仅含有一个理论上可修饰位点的肽段,卤化位点的精确定位仍然需要修饰残基两侧骨架碎裂产生的丰富碎片离子以提高修饰位点定位的可信度。如图5所示,193 nm UVPD相比于HCD解离模式观察到了Br丢失的肽段自由基质谱峰,在色氨酸的周围骨架发生了广泛的解离并提供了丰富的碎片离子,从而提供了更高的Xcorr值。在HCD解离图谱中色氨酸和亮氨酸之间没有鉴定到骨架碎裂产生的N端或C端碎片离子(图5a)。相比之下,在该肽段的193 nm UVPD解离图谱中鉴定到了对应于色氨酸残基两侧骨架裂解的N端碎片离子峰a_4_、b_4_、c_4_和C端碎片离子峰的y_5_、y_6_-H_2_O、z_6_、z_6_-1(图5b)。其中a_4_、b_4_、c_4_、y_5_的单同位素质量没有溴氧化修饰的偏移,而y_6_-H_2_O、z_6_、z_6_-1的单同位素质量发生了溴氧化修饰的偏移,实现了溴氧化修饰位点的精确定位。

**图 5:**
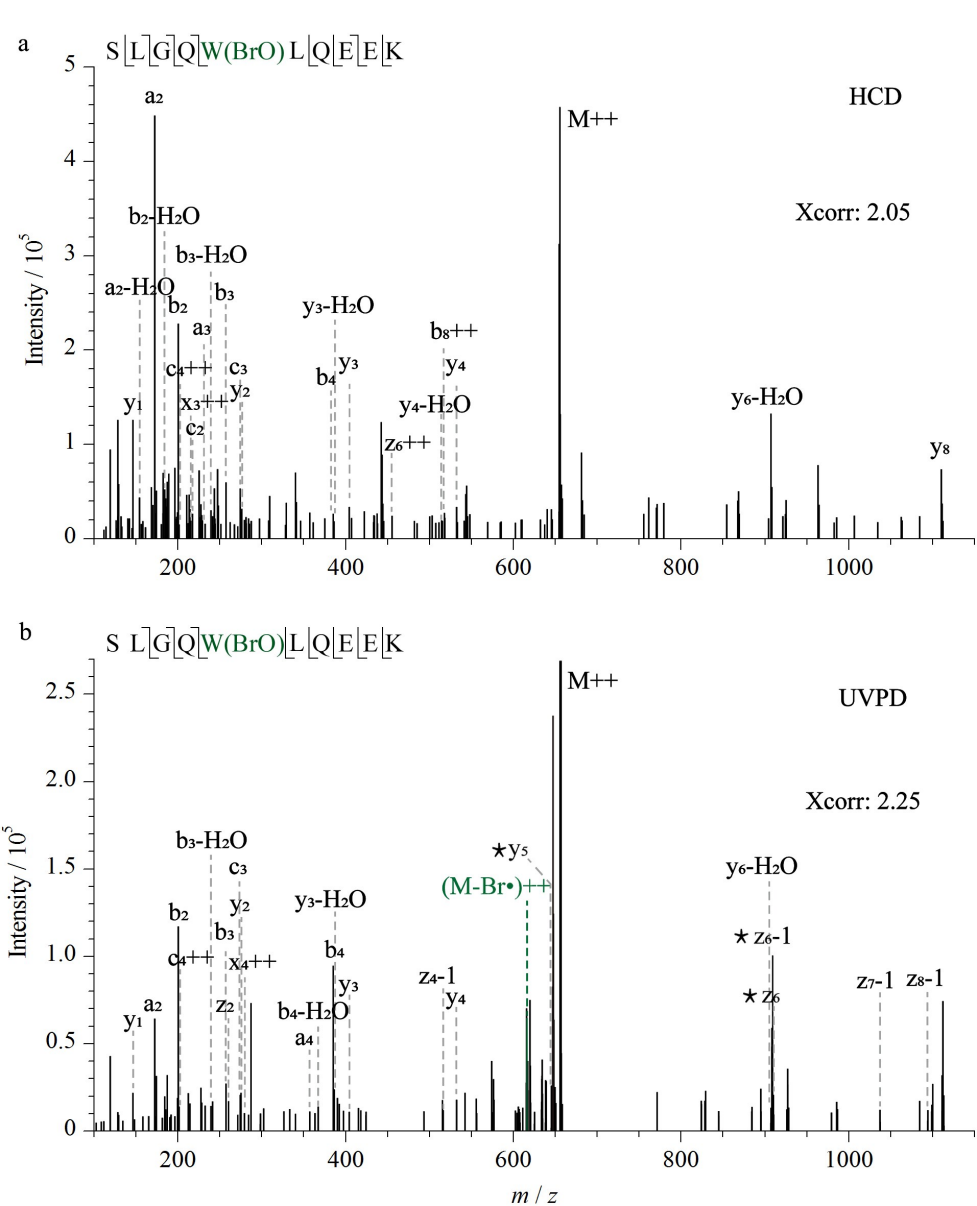
溴化SLGQWLQEEK肽段经(a)HCD和(b)193 nm UVPD解离模式鉴定的二级质谱图

## 3 结论

本研究利用光化学标记方法实现了鼠肝酶解肽样品的高效率溴化和碘化修饰,并通过193 nm UVPD结合常规的HCD解离模式对光化学溴化和碘化标记后的组学样品进行了光解离机制和序列、修饰位点分析。卤化修饰赋予了肽段独特的光切割标签,在真空条件下利用193 nm高能紫外激光激发卤化肽段引发了新型自由基解离途径,卤化位点的C-Br键或C-I键断裂产生多肽自由基离子,通过自由基的迁移进一步引起多肽骨架碎裂产生Br、I丢失的碎片离子。组学分析发现常规的HCD解离模式结合193 nm UVPD可以提高鼠肝酶解肽卤化标记后的位点定位准确性。这种光化学溴化、碘化修饰和193 nm UVPD高效整合的新型蛋白质组学质谱分析策略在蛋白质组学分析中具有广阔的应用前景。
